# Metabolomics Analysis of the Effect of Glutamic Acid on Monacolin K Synthesis in *Monascus purpureus*

**DOI:** 10.3389/fmicb.2020.610471

**Published:** 2020-12-17

**Authors:** Chan Zhang, Nan Zhang, Mengxue Chen, Haijiao Wang, Jiachen Shi, Bei Wang, Baoguo Sun, Chengtao Wang

**Affiliations:** ^1^Beijing Advanced Innovation Center for Food Nutrition and Human Health, Beijing Technology & Business University, Beijing, China; ^2^Beijing Engineering and Technology Research Center of Food Additives, Beijing Technology & Business University, Beijing, China

**Keywords:** monacolin K, monascus, metabolomics, citric acid, glutamic acid

## Abstract

Monacolin K is a secondary metabolite produced by *Monascus* with beneficial effects on health, including the ability to lower cholesterol. We previously showed that the yield of monacolin K was significantly improved when glutamic acid was added to the fermentation broth of *Monascus purpureus* M1. In this study, we analyzed *M. purpureus* in media with and without glutamic acid supplementation using a metabolomic profiling approach to identify key metabolites and metabolic pathway differences. A total of 817 differentially expressed metabolites were identified between the two fermentation broths on day 8 of fermentation. Pathway analysis of these metabolites using the KEGG database indicated overrepresentation of the citric acid cycle; biotin metabolism; and alanine, aspartate, and glutamate metabolic pathways. Six differentially expressed metabolites were found to be related to the citric acid cycle. The effect of citric acid as an exogenous additive on the synthesis of monacolin K was examined. These results provide technical support and a theoretical basis for further studies of the metabolic regulatory mechanisms underlying the beneficial effects of monacolin K and medium optimization, as well as genetic engineering of *Monascus* M1 for efficient monacolin K production.

## Introduction

*Monascus* is a common saprophytic fungus with practical applications in the food, brewing, and medical industries in China (Lin et al., 2008; Pérez-Jiménez et al., 2018). *Monascus* species, such as *M. ruber, M. fuliginosus, M. albidus, M. rubiginosus, M. serorubescens*, and *M. purpureus* are widely known to produce various secondary metabolites with polyketide structures, such as pigments (Krairak et al., 2000), monacolin K (Endo, 1979), citrinin (Blanc et al., 1995), and γ-aminobutyric acid (Diana et al., 2014). Extensive studies of physiological substances in *Monascus* have led to the discovery of various metabolites with high nutritional and pharmaceutical value (Li et al., 2011; Stefanutti et al., 2017).

Among these metabolites, monacolin K is widely used as a drug for the treatment of hyperlipidemia (Anagnostis et al., 2018; Lee et al., 2018). Monacolin K can effectively suppress the activity of a key enzyme in cholesterol biosynthesis (HMG-CoA) as a competitive inhibitor and can regulate blood lipid abnormalities (Su et al., 2003). Moreover, it can suppress breast cancer cell proliferation (Patel, 2016) and facilitate apoptosis in malignant thyroid cells (Chen et al., 2014). However, its use is limited by low yields and high production costs. Therefore, it is important to improve the production of monacolin K by *Monascus*. Generally, the yield of monacolin K is improved by two approaches. First, high monacolin K-producing strains can be produced by genetic engineering technology (Liu et al., 2019) and mutation breeding (Chen et al., 2008). Second, fermentation conditions of *Monascus*, such as medium components and conditions, can be optimized (Kalaivani and Rajasekaran, 2014). The regulatory mechanism of monacolin K has not been fully resolved. Metabolomics techniques are used to analyze the differences in key metabolites involved in the synthesis of monacolin K, which is a better analytical method.

Metabolomics is a new qualitative and quantitative method for comprehensive analyses of small molecule metabolites (<1.5 kDa) produced by an organism in a specific physiological period (Zhang et al., 2016); this approach is widely used in the food, medical, and agricultural fields (Vongsangnak et al., 2011; Johanningsmeier et al., 2016). Metabolites serve as direct indicators of biological activity and are therefore, useful for detecting correlations with phenotypes (Patti et al., 2012). Both targeted (Dudley et al., 2010) and untargeted metabolomics approaches can characterize metabolites that accumulate differentially in biological samples (Nordström et al., 2008). Filamentous fungi produce numerous secondary metabolites that are not directly involved in growth or reproduction (Keller et al., 2005). Increasing studies have focused on the characterization of filamentous fungi by metabolite profiling and metabolomics. Liquid chromatography high-resolution mass spectrometry (LC-HRMS) and gas chromatography mass spectrometry (GC-MS) were used by (Kang et al., 2011) to categorize *Trichoderma* species according to their secondary metabolite profiles (Kang et al., 2011). Identifying unknown bioactive compounds produced by filamentous fungi is also a major research goal (Wiemann et al., 2013). Several compounds that are correlated with co-cultivation of *Streptomyces coelicolor* and *Aspergillus niger* have been identified by nuclear magnetic resonance (NMR)-based metabolomics (Wu et al., 2015). A variety of volatile organic compounds associated with the catabolism of branched chain amino acids have been detected by solid phase microextraction (SPME)-GC-MS (Roze, 2010). Major differences between *Escherichia coli* strains grown under different conditions have been examined at the levels of amino acids, fatty acids, and precursor metabolites by GC-MS (Carneiro et al., 2012).

Despite this increase in metabolomics studies of filamentous fungi, this technology has not been used to investigate changes in the metabolome of *Monascus* during different growth phases. Previous analyses showed that glutamic acid can enhance the production of monacolin K in *Monascus* M1 (Zhang et al., 2017). In this study, the fermentation broths prepared using two types of medium were collected at different time points for metabolomic profiling by ultra-performance liquid chromatography quadrupole time-of-flight mass spectrometry (UPLC-Q-TOF-MS). The composition of metabolites and molecular mechanism by which glutamic acid regulates monacolin K production were investigated.

## Methods and Materials

### Fungal Strain and Culture Conditions

*Monascus purpureus* M1 was obtained from the Chinese General Microbiological Culture Collection Center (strain number, CGMCC 3.0568, Beijing, China). *M. purpureus* M1 is a wild-type strain that stably produces monacolin K. It was grown on potato dextrose agar at 30°C for 4 days and cultured with 50 mL of seed medium containing 30 g/L glucose, 15 g/L soybean powder, 1 g/L MgSO_4_·7H_2_O, 2 g/L KH_2_PO_4_, 70 g/L glycerol, 2 g/L NaNO_3_, and 10 g/L peptone at neutral pH. The cultures were incubated at 30°C for 48 h with shaking at 200 rpm. Two types of fermentation medium were used. The original fermentation medium contained 20 g/L rice powder, 1 g/L MgSO_4_·7H_2_O, 2 g/L ZnSO_4_·7H_2_O, 2.50 g/L KH_2_PO_4_, 90 g/L glycerol, 5 g/L NaNO_3_, and 10 g/L peptone at a neutral pH. For the glutamic acid fermentation medium, the original fermentation medium was supplemented with 10 mM glutamic acid. The seed culture (5 mL) was then inoculated into these two types of fermentation media (50 mL). The cultures were incubated at 30°C for 2 days with shaking at 150 rpm, followed by incubation at 25°C for 10 days with shaking at 150 rpm.

### Determination of Monacolin K

To evaluate the yield of monacolin K, the fermentation broth (5 mL) was added to 15 mL of 75% methanol (v/v) and sonicated for 20 min, and the supernatant was passed through a 0.45 μm filter. High-performance liquid chromatography (HPLC) using an Inertsil ODS-3 C18 column (150 mm × 4.6 mm × 5 μm) was used to detect the yield. The mobile phase was ddH_2_O (with 0.1% H_3_PO_4_) /methanol (1:3, v/v) and was run at 1 mL/min. An ultraviolet detector was used at a wavelength of 237 nm, detection temperature of 30°C, and injection volume of 10 μL.

### Sample Collection and Preparation

The fermentation medium was collected at 0, 8, and 12 days in two different cultures and stored at−80°C until analysis. NG0, NG8, and NG12 indicate the fermentation medium without glutamic acid on days 0, 8, and 12, respectively; G8 and G12 indicate the fermentation medium with glutamic acid on days 8 and 12. A volume of 300 μL of 80% methanol was added to 100 μL of each sample followed by ultrasonication for 10 min; ultrasonication was stopped for 10 s every 5 s. The samples were vortexed for 1–3 min and left standing for 10 min at 4°C. To separate the methanol/water layers, the samples were centrifuged for 10 min at 13,000 rpm. The supernatant was dried to a powder by vacuum drying and dissolved in methanol.

### UPLC-Q-TOF-MS

Liquid chromatography (LC) separation was performed using an Agilent ZORBAX Eclipse Plus C18 column (100 × 2.1 mm, 3.5 μm; Agilent Technologies, Santa Clara, CA, USA). The injected sample volume was 20 μL for each run in the full loop injection mode, and the column temperature was 25°C. The flow rate of the mobile phase was 0.5 ml/min, where mobile phases A and B were 0.1% formic acid in ddH_2_O and acetonitrile, respectively. The program for elution gradient is described in Table 1.

**Table 1:** Gradient elution conditions in LC.

**Time (min)**	**Mobile phase A (%)**	**Mobile phase B (%)**
0	98	2
1	98	2
13	10	90
16	10	90
16.1	98	2
20	98	2

MS was performed using Triple TOF 5600+, an orthogonal accelerated TOF-MS (AB Sciex, Redwood City, CA, USA) equipped with an electrospray ion source. Data were acquired in positive and negative-V-geometry mode for each LC-MS analysis. The capillary voltages were set to 2,500 and 3,000 V, cone gas flow rate 50 L/h, desolvation gas flow rate 600 L/h, source temperature 120°C, and desolvation temperature 500°C. The scan range of mass-to-charge (m/z) was 50 to 1,500 in full scan mode and data were collected in centroid mode. Independent reference lock-mass ions obtained by Analyst TF 1.6 and MarkerView 1.2.1 were used to ensure mass accuracy during data acquisition.

The differentially expressed metabolite ions were identified by searches against the HMDB (http://www.hmdb.ca/spectra/ms/search) databases (Wishart et al., 2008). The mass tolerance for the HMDB database search was set to 0.05 Da. The chromatographic retention behavior was also considered in order to reduce false-positive matches.

### Statistical Analysis

MarkerView was used for peak identification, peak filtering, and peak alignment of raw mass spectrometry data. The qualitative m/z and a two-dimensional data matrix of peak areas were obtained. MetaboAnalyst 3.0 was used to normalize samples with different requirements for comparison. Differentially expressed metabolites among groups were visualized by principal component analysis (PCA), partial least squares discriminant analysis (PLS-DA), and orthogonal partial least squares-discriminant analysis (OPLS-DA). The false discovery rate method was used to correct for multiple comparisons. Data are presented as the mean ± SD.

Multivariate analyses, including unsupervised PCA and supervised PLS-DA, were implemented in MetaboAnalyst 4.0 (http://www.metaboanalyst.ca/MetaboAnalyst/). PLS-DA models were cross-validated by the 10-fold method with unit variance scaling. The parameter *R*^2^ was used to evaluate the fitting of the PLS-DA models, and *Q*^2^ was used to assess predictive ability. Negative or very low *Q*^2^ values indicate that the differences between groups were not significant. The PLS-DA model removes variation in the *X* matrix that is not correlated with the *Y* matrix. Thus, only one predictive component is generally used for discrimination between two classes.

Comparisons in the intensities of integrated regions between two groups were performed using the two-tailed Welch's *t*-test implemented in MetaboAnalyst 4.0, and *p* < 0.05 was considered statistically significant. A volcano plot was generated based on a combination of fold change values and *t*-tests, and significantly different peaks among the three groups were used for multivariate pattern recognition. Moreover, peaks that were consistently upregulated or downregulated were identified; the intensity data for these regions were used in box-plot, hierarchical cluster, and metabolic pathway analyses.

### Pathway Analysis

Differences in chemical metabolites were evaluated using the MetaboAnalyst 4.0 web portal for pathway analysis and visualization (http://www.metaboanalyst.ca/). Additional metabolite set enrichment analysis was performed (http://www.metaboanalyst.ca/). Pearson's correlation coefficients were calculated to evaluate the relationships between biomarkers (*p* < 0.05, impact >0.01).

### Verification of Fermentation Experiment

Fermentation experiments were carried out on the characteristic substances selected by the metabolome to verify their functions. According to the compounds involved in the tricarboxylic acid cycle, this study chose to add malic acid, fumaric acid, α-ketoglutarate, and citric acid to the common medium. Based on the preliminary exploration of the optimal concentration in our laboratory, the concentration of the above-mentioned substances was determined. For the malic acid-fermentation medium, fumaric acid-fermentation medium, α-ketoglutarate-fermentation medium, and citric acid-fermentation medium, the original fermentation medium was separately supplemented with 3 g/L malic acid, 0.60 g/L fumaric acid, 10 g/L α-ketoglutarate, 1 g/L citric acid. The culture conditions were the same as 2.1.

## Results

### Glutamic Acid Influences Monacolin K Yield

**Figure 1:**
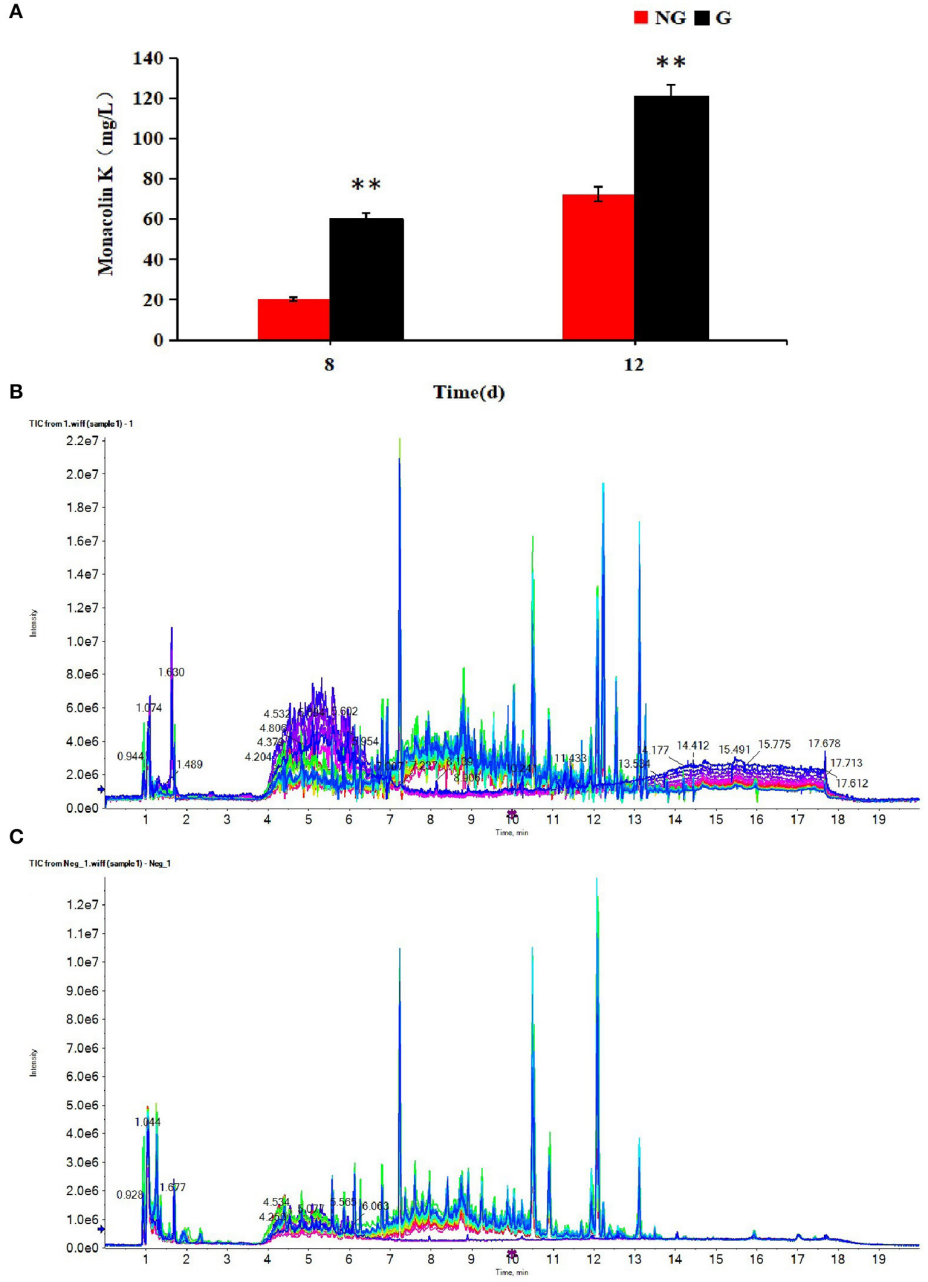
**(A)** Effect of glutamic acid on monacolin K production in *Monascus*, **(B)** the TIC diagrams of positive, **(C)** and negative ions for all samples. **(B,C)** Indicate that all samples were available. **p < 0.01.

Monacolin K production was detected at 8 and 12 days in two different cultures. As shown in Figure 1A, the monacolin K yield using the M1 strain was higher in a glutamic acid-containing medium than in an ordinary medium on days 8 and 12, with increases of 2.90 and 1.70-fold, respectively.

### Multivariate Statistical Analysis

The profiles of metabolites in medium detected by UPLC-Q-TOF-MS were analyzed by multivariate statistical methods, including PCA, PLS-DA, and OPLS-DA. The TIC diagrams of all samples in positive and negative ion mode are shown in Figures 1B,C.

**Figure 2:**
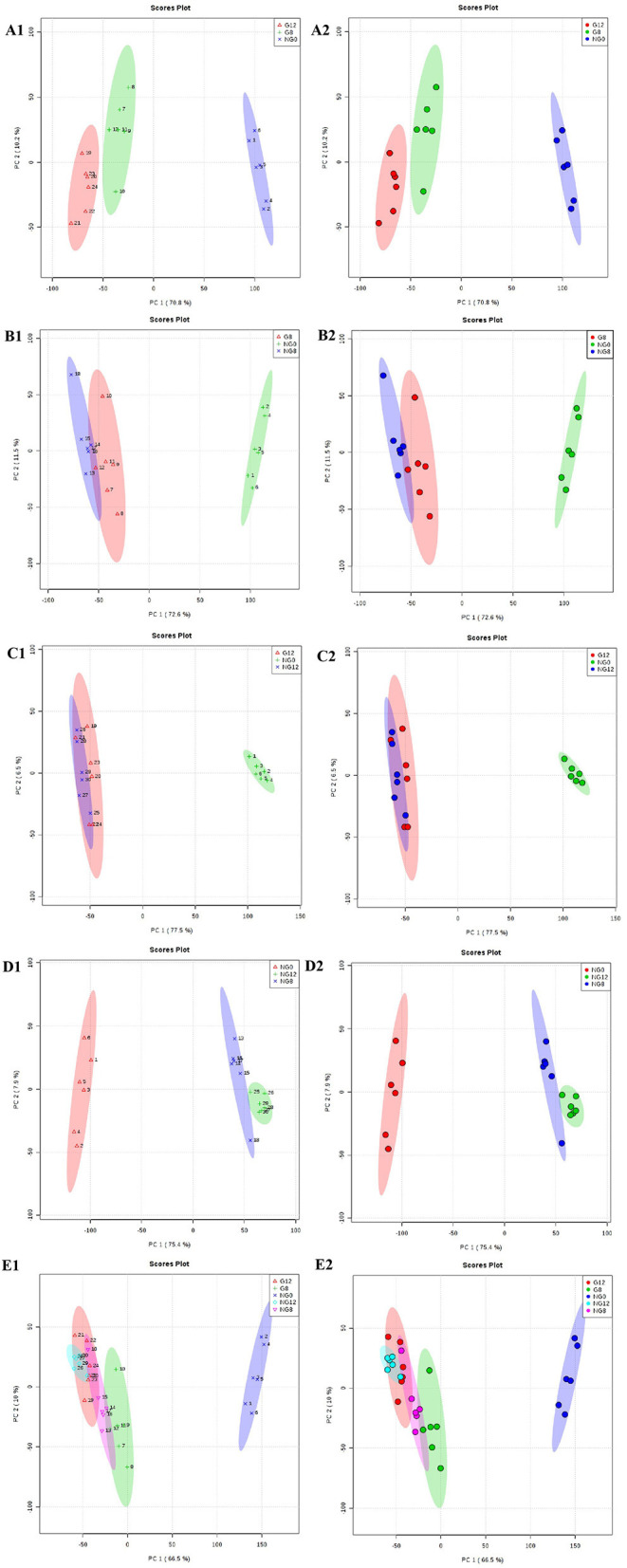
PCA score plots. **(A1,A2)** NG0-G8-G12; **(B1,B2)** NG0-G8-NG8; **(C1,C2)** NG0-G12-NG12; **(D1,D2)** NG0-NG8-NG12; **(E1,E2)** G8-G12-NG0-NG8-NG12. The middle point represents the sample number, and the contribution rate of PC1 and PC2 in the score chart is shown. The area within the middle ellipse represents the 95% confidence interval.

Through PCA analysis, inspect the distribution of samples, verify the rationality of the experimental design and the uniformity of biological replicate samples. The PCA analysis results were shown in Figure 2. The middle point in Figure 2 represents the sample number, and the contribution rate of PC1 and PC2 in the score chart is shown in Figure 2. The area within the middle ellipse represents the 95% confidence interval. It could be seen from Figure 2 that the samples of the same group were relatively concentrated in two-dimensional space, indicating that the selection of these indicators was representative and the biological repetition was good.

**Figure 3:**
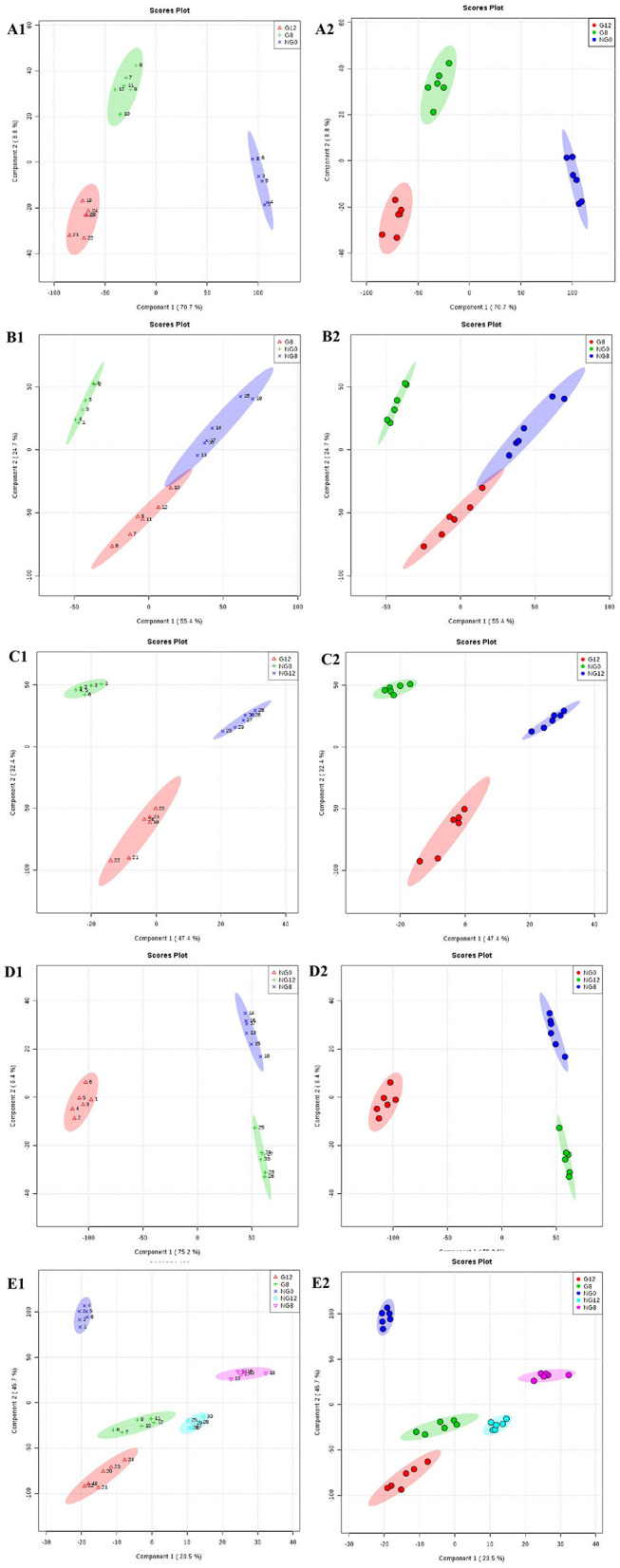
PLS-DA score plots. **(A1,A2)** NG0-G8-G12; **(B1,B2)** NG0-G8-NG8; **(C1,C2)** NG0-G12-NG12; **(D1,D2)** NG0-NG8-NG12; **(E1,E2)** G8-G12-NG0-NG8-NG12. The contribution rate of PC1 and PC2 in the score chart is shown. The area inside the middle ellipse represents the 95% confidence interval.

The PLS-DA results are shown in Figure 3; this analysis was performed to visualize the differentiation among the three groups (NG0, G8, and G12) (Figures 3A1,A2). The selection of indicators was representative, and repeatability among biological replicates was acceptable. Components within groups were relatively concentrated, and the NG0-G8-NG8 groups (Figures 3B1,B2), NG0-G12-NG12 groups (Figures 3C1,C2), NG0-NG8-NG12 groups (Figures 3D1,D2), and G8-G12-NG0-NG8-NG12 groups (Figures 3E1,E2) showed similar results. The contribution rate of PC1 and PC2 in the score chart was shown in Figure 3. The area inside the middle ellipse represents the 95% confidence interval.

**Figure 4:**
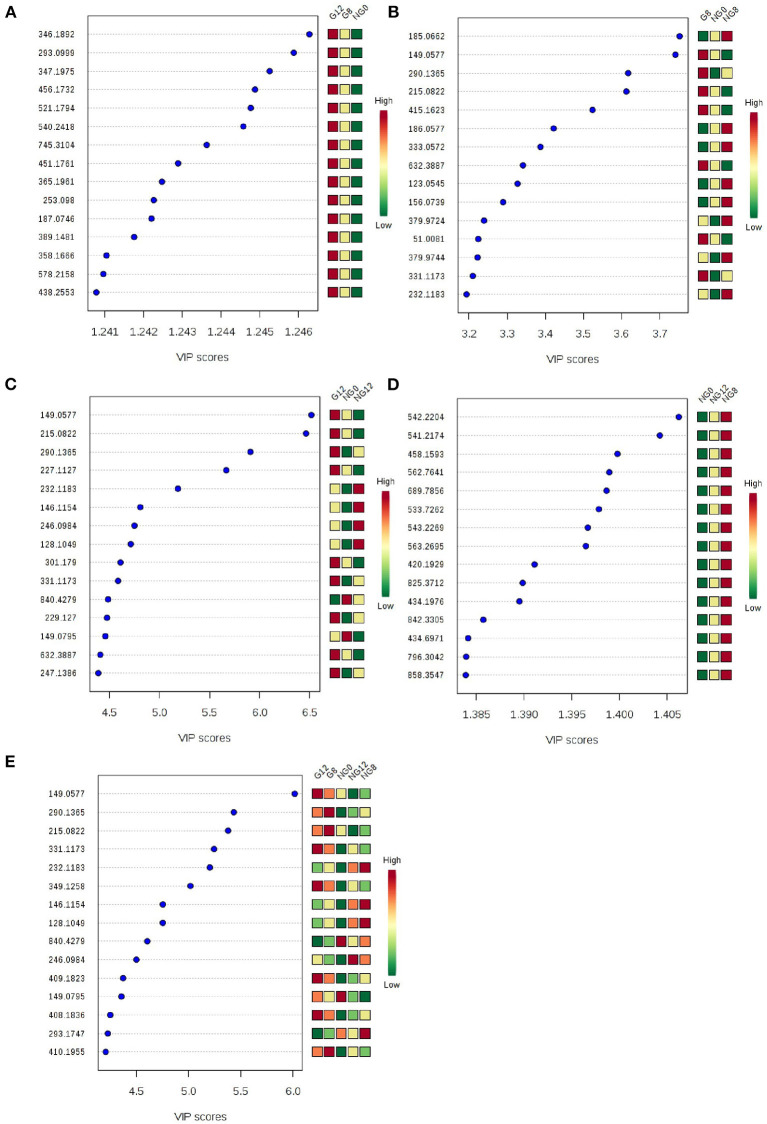
PLS-DA-vip score of samples. **(A)** NG0-G8-G12; **(B)** NG0-G8-NG8; **(C)** NG0-G12-NG12; **(D)** NG0-NG8-NG12; **(E)** G8-G12-NG0-NG8-NG12. Part of the molecular weight list of VIP >1, the abscissa represents the VIP score value, the ordinate represents the molecular weight information of m/z, and the red and green on the right represent the expression level of m/z in the two groups.

At the same time, VIP value (variable importance in projection) was a variable importance factor. Generally, VIP >1 can be considered as a difference. The PLS-DA-vip score as shown in Figure 4. Part of the molecular weight list of VIP >1, the abscissa represents the VIP score value; the ordinate represents the molecular weight information of m/z. Figures 4A–E showed that all the samples were different.

**Figure 5:**
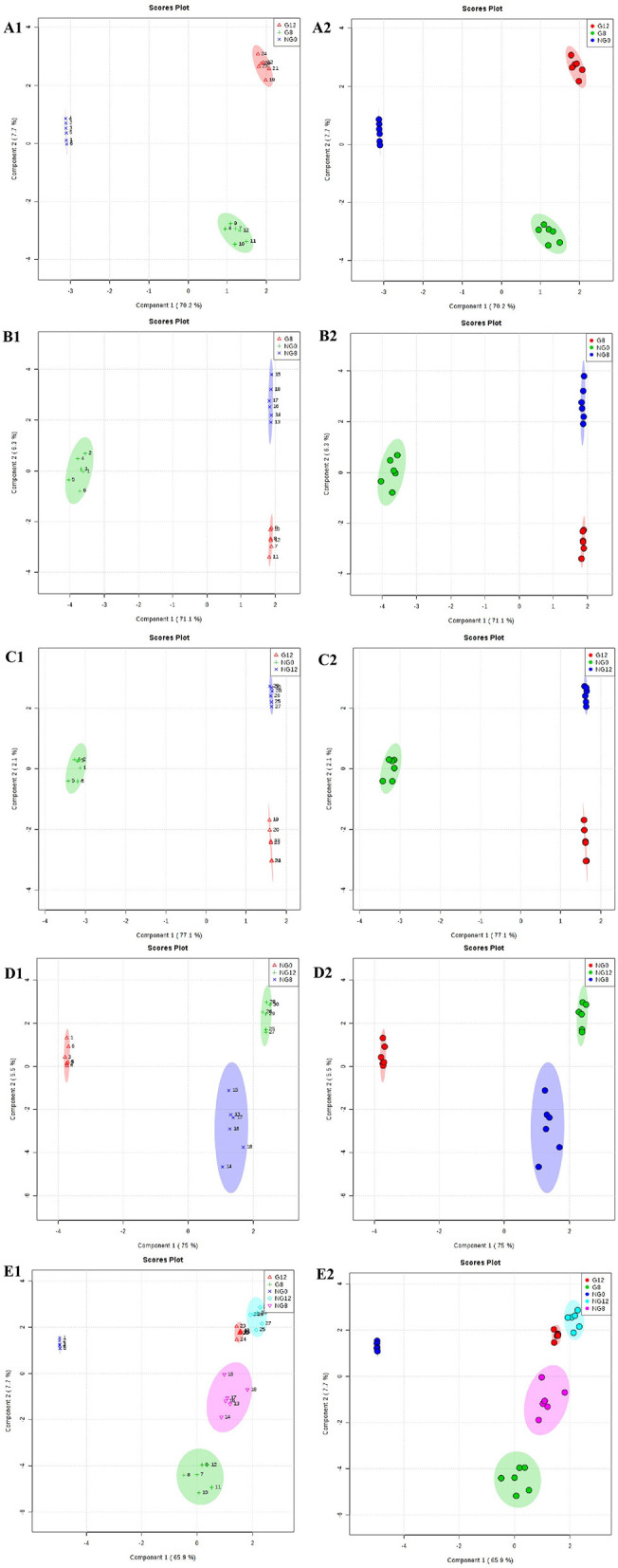
OPLS-DA score plots for the data sets. **(A1,A2)** NG0-G8-G12; **(B1,B2)** NG0-G8-NG8; **(C1,C2)** NG0-G12-NG12; **(D1,D2)** NG0-NG8-NG12; **(E1,E2)** G8-G12-NG0-NG8-NG12. The OPLS-DA model shows significant differences among the different groups.

Furthermore, OPLS-DA was used to filter uncorrelated signals related to model classification. As shown in Figures 5E1,E2, OPLS-DA models showed a distinct separation between NG0-NG8-NG12-G8-G12 groups. Accordingly, there were significant differences among groups, and six repeated data points in each group were highly aggregated, indicating good repeatability. In addition, the components for different groups were separated with significant differences (Figures 5A–D).

### Difference Analysis

**Figure 6:**
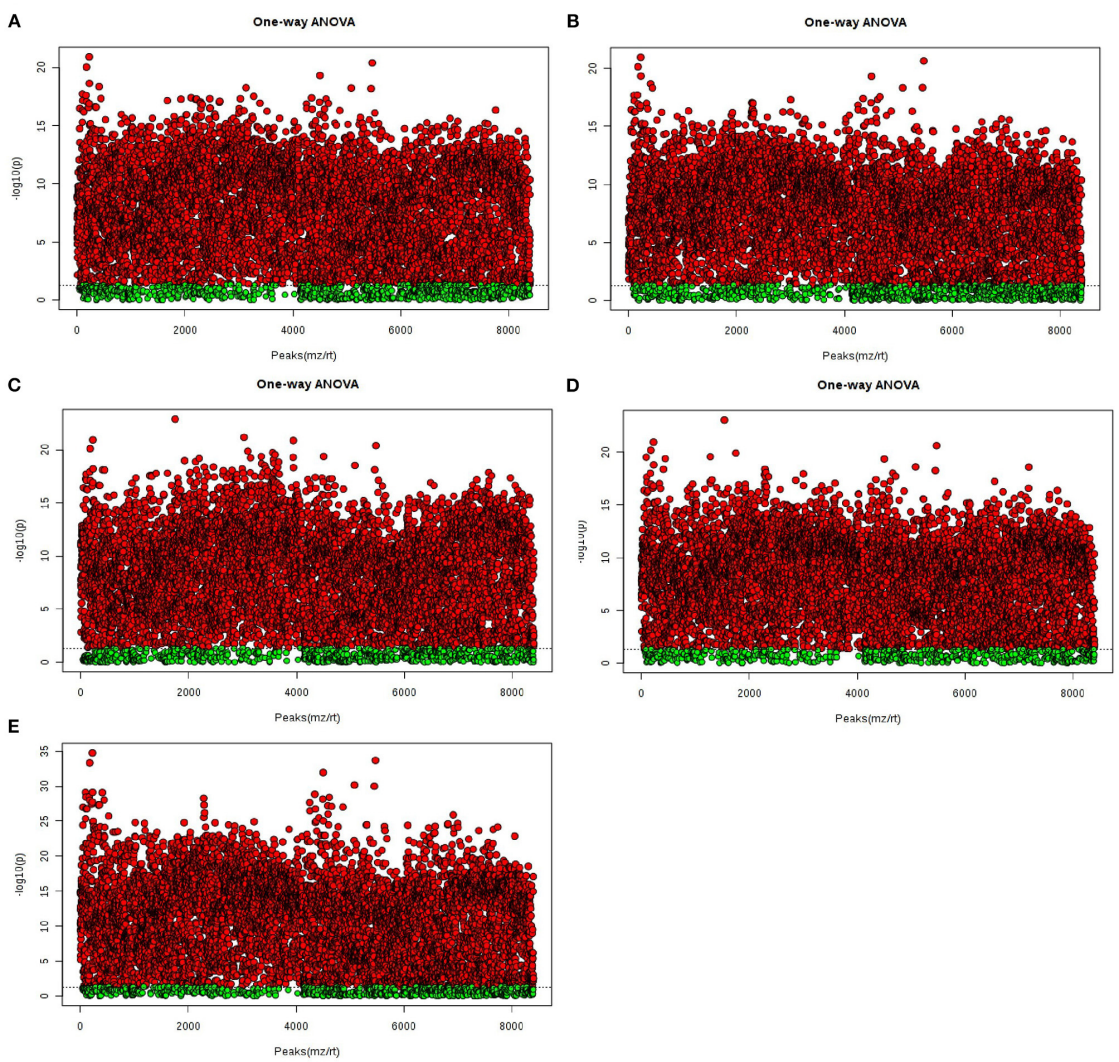
The ANOVA volcano maps of samples. **(A)** NG0-G8-G12; **(B)** NG0-G8-NG8; **(C)** NG0-G12-NG12; **(D)** NG0-NG8-NG12; **(E)** G8-G12-NG0-NG8-NG12. The red point represents *p* < 0.05, which means there is a significant difference.

In the analysis of ANOVA volcano graph, the *p*-value needed to be considered at the same time. After the ANOVA test data was converted into a negative logarithm base 10, the graph was shown in Figure 6. The NG0-G8-G12 groups (Figure 6A), NG0-G8-NG8 groups (Figure 6B), NG0-G12-NG12 groups (Figure 6C), NG0-NG8-NG12 groups (Figure 6D), and G8-G12-NG0-NG8-NG12 groups (Figure 6E) showed similar results. The red point represented *p* < 0.05, which meant there was a significant difference.

**Figure 7:**
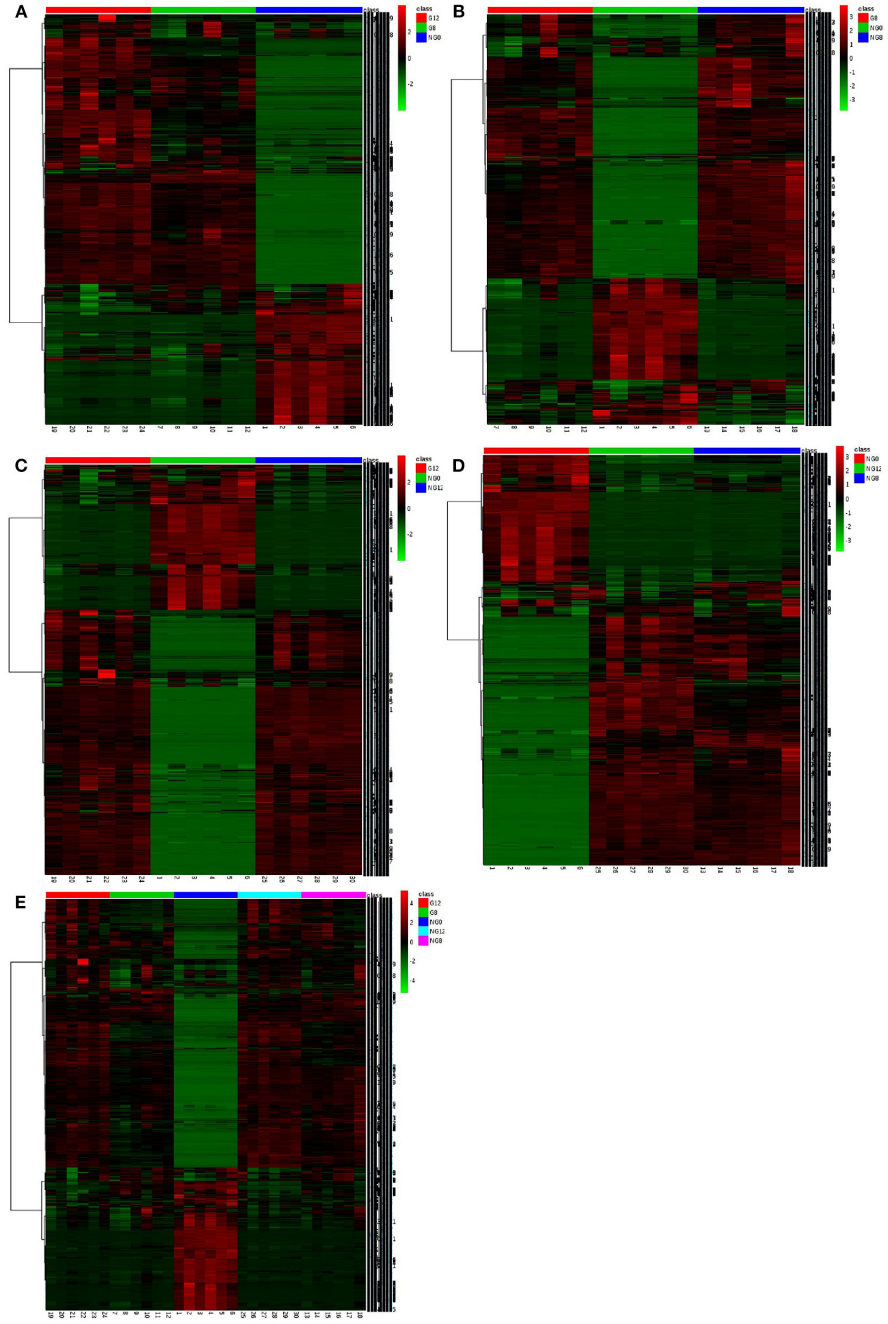
The cluster maps of samples. **(A)** NG0-G8-G12; **(B)** NG0-G8-NG8; **(C)** NG0-G12-NG12; **(D)** NG0-NG8-NG12; **(E)** G8-G12-NG0-NG8-NG12. The abscissa represents the sample number, and the ordinate represents the molecular weight of each metabolite.

In the cluster analysis, all the metabolites obtained were subjected to two-way clustering of samples and metabolites, and the method used was hierarchical clustering. In Figure 7, the abscissa represented the sample number, and the ordinate represented the molecular weight of each metabolite. From the results shown in Figure 7A, it can be seen that G12 and G8 have similar metabolic profiles and were clustered together. G8-NG8 (Figure 7B), G12-NG12 (Figure 7C), NG12-NG8 (Figure 7D), and G12-G8-NG12-NG8 (Figure 7E) also had similar metabolic profiles.

### Identification of Metabolites

**Figure 8:**
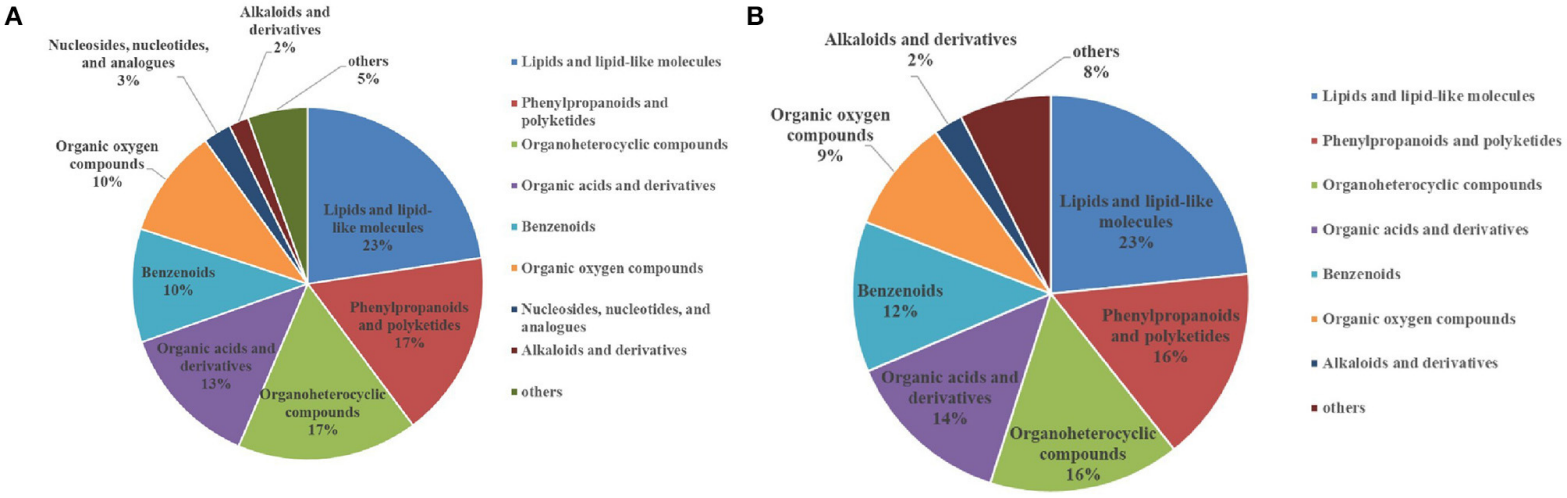
Superclass pie chart of differential metabolites between NG8 and G8 **(A)** and between NG12 and G12 **(B)**.

After analysis of the metabolomics profiles of different groups, differential metabolites were selected based on VIP scores. A total of 2,509 differential metabolites were detected between NG8 and G8. Through the use of a fold-change >2, VIP >1, and *p* < 0.05, 817 differentially expressed metabolites were selected. More than 18 differential metabolites were screened according to superclass classification. A superclass pie chart of differential metabolites between NG8 and G8 is shown in Figure 8A. The metabolites included “lipids and lipid-like molecules” (*n* = 185, 23%); “phenylpropanoids and polyketides” (*n* = 140, 17%); “organoheterocyclic compounds” (*n* = 136, 17%); “organic acids and derivatives” (*n* = 108, 17%); “benzenoids” (*n* = 85, 10%); and “organic oxygen compounds” (*n* = 82, 10%).

A total of 2,488 differential metabolites was detected between NG12 and G12. Using a fold-change > 2, VIP > 1, and *p* < 0.05 as criteria, 628 differential metabolites were selected. More than 17 types of differential metabolites were screened according to superclass classification. A superclass pie chart of differential metabolites between NG12 and G12 is shown in Figure 8B. The metabolites included “lipids and lipid-like molecules” (*n* = 147, 23%); “phenylpropanoids and polyketides” (*n* = 100, 16%); “organoheterocyclic compounds” (*n* = 98, 16%); “organic acids and derivatives” (*n* = 77, 14%); “benzenoids” (*n* = 77, 12%); and “organic oxygen compounds” (*n* = 58, 9%).

A total of 817 differential metabolites were putatively identified (VIP > 1, *p* < 0.05) between NG8 and G8 and subsequently categorized in subclasses, including fatty acids and conjugates, amino acids, peptides, and analogs. Six differential metabolites were classified as fatty acids and conjugates (Table 2). A variety of metabolites were produced during the growth of microorganisms. Among them, fatty acids and conjugates were very common. Molds, bacteria, yeast, and some algae can produce lipid metabolites. Particularly, molds can produce many types of fatty acids, including many beneficial polyunsaturated fatty acids. *Monascus* is rich in polyunsaturated fatty acids, and unsaturated fatty acids may enhance the cholesterol-inhibiting effects of monacolin K. Fatty acids in *Monascus* mainly include C18:1, C18:2, C16:0, C18:0, and C16:1 (Dr et al., 1989). The pathway for the production of polyketide secondary metabolites by *Monascus* is related to the fatty acid production pathway because acetyl-CoA is a common initial substance for these two pathways. Acetyl-CoA is synthesized in the early fermentation period of *Monascus* and acetyl-CoA is mainly used to synthesize lipid compounds. At the end of fermentation, the fatty acid contents were low; acetyl-CoA is mainly used to synthesize *Monascus* pigments (Somashekar and Joseph, 2000). As shown in differential metabolites of fatty acids and conjugates among NG0-G8-NG8 (Table 2), the contents of petroselinic acid and oleic acid decreased following the addition of glutamic acid. Acetyl-CoA was presumably mainly used to synthesize secondary metabolites in *Monascus*.

**Table 2:** Differential metabolites of fatty acids and conjugates among NG0-G8-NG8.

**Compound name**	**Query mass**	**VIP**	**Formula**	**Peak area in NG0**	**Peak area in G8**	**Peak area in NG8**
3-Dehydroxycarnitine	146.1154	2.5433	C_7_H_15_NO_2_	55,558	97,272	121,302
Mevalonic acid	149.0795	2.3703	C_6_H_12_O_4_	239,155	269,756	126,102
2-Hydroxyadipic acid	161.0456	1.6775	C_6_H_10_O_5_	1,057,921	798,989	694,349
Petroselinic acid	280.2348	1.8957	C_18_H_33_O_2_	901	19,043	37,173
Oleic acid	281.2466	1.8399	C_18_H_34_O_2_	6737	13,077	29,831
12-Oxo-20-carboxy-leukotriene B4	363.18	2.447	C_20_H_28_O_6_	17,560	34,749	78,353

### KEGG Pathway Analysis of Differential Metabolites

**Figure 9:**
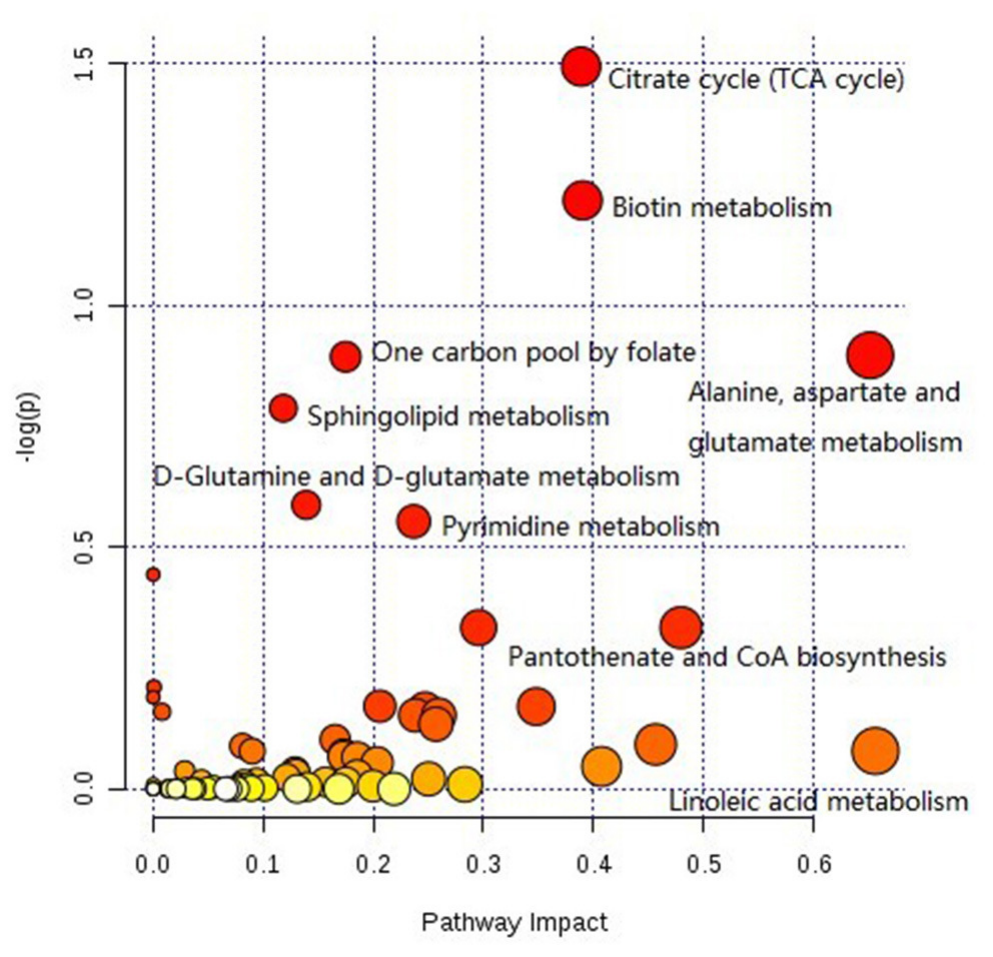
KEGG pathway analysis diagram.

The pathway analysis of differential metabolites was performed using the KEGG database. As shown in Figure 9, many metabolic pathways were identified in this analysis. Among them, citric acid cycle, biotin metabolism, alanine, aspartate, and glutamate metabolism, one carbon pool by folate, sphingolipid metabolism, d-glutamine and d-glutamate metabolism, pyrimidine metabolism, d-arginine and d-ornithine metabolism, valine, leucine, isoleucine biosynthesis, pantothenate, and CoA biosynthesis were potential target pathways with a high impact and low false discovery rate.

### Fermentation Test Results

**Figure 10:**
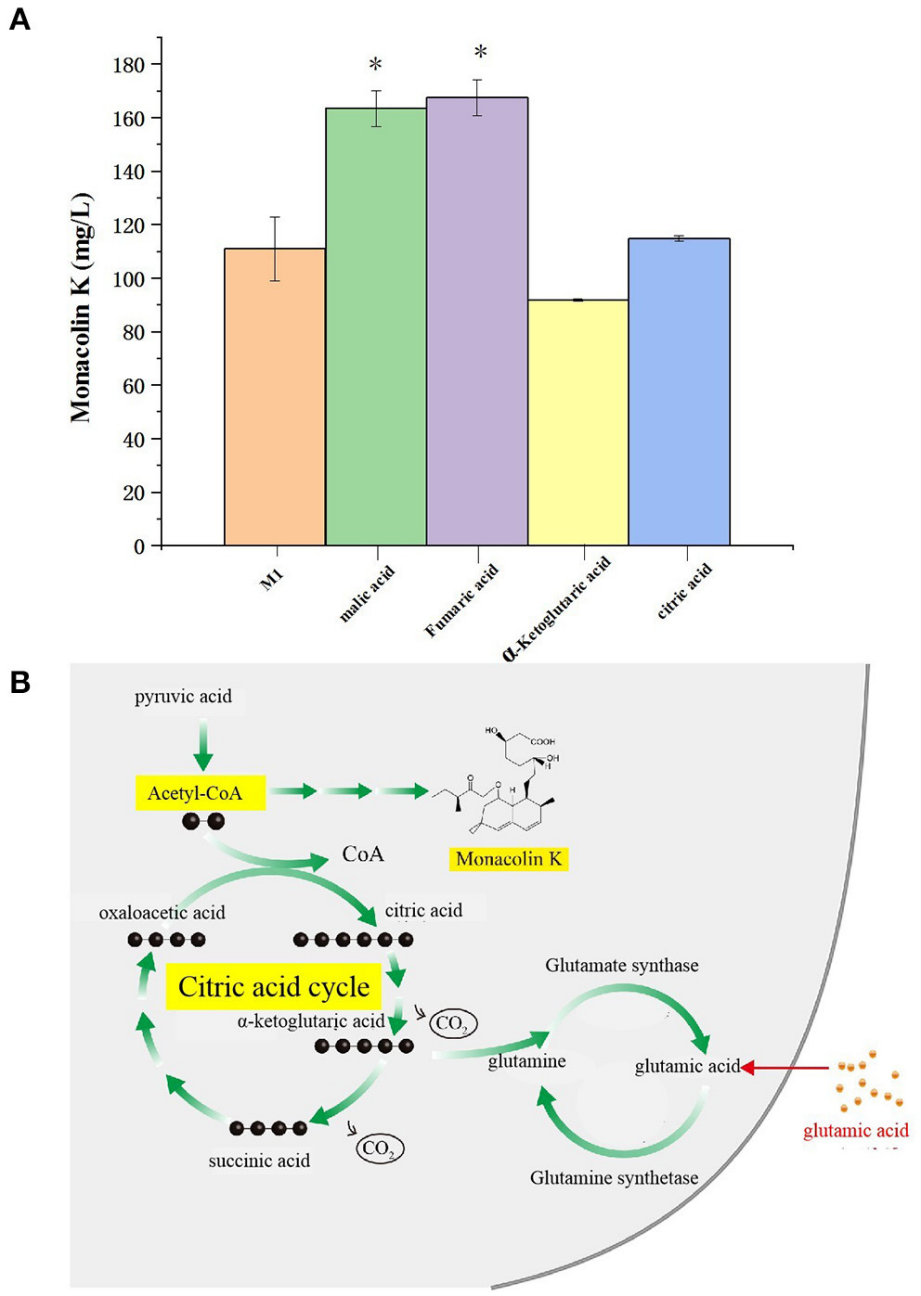
**(A)** Effect of differential metabolite on the yield of monacolin K (* represents *p* < 0.05) and **(B)** schematic diagram to summarize the proposed model for the relationship among the citric acid cycle, acetyl-CoA, monacolin K, and any other metabolites.

The citric acid cycle is a cyclic reaction system composed of a series of enzymatic reactions, with the condensation reaction of acetyl-CoA and oxaloacetic acid as the initial reaction. Because acetyl-CoA is the common key substance of the citric acid cycle and monacolin K synthesis, it is speculated that the regulatory pathways of the citric acid cycle and monacolin K influence each other to a large extent. Therefore, we combined the differential metabolites obtained through metabolomics with the compounds involved in the citric acid cycle as screening conditions, and we selected malic acid, fumaric acid, α-ketoglutarate, and citric acid for fermentation experiment verification. In order to verify the above speculation, malic acid, fumaric acid, α-ketoglutarate, and citric acid were added to the original medium, and the change in the production of monacolin K cultured in the original medium was detected. Figure 10A shows that malic acid, fumaric acid, and citric acid have a promoting effect on the production of monacolin K. Among them, malic acid and fumaric acid had significant effects, and α-ketoglutarate inhibited the production of monacolin K. It was speculated that the accumulation of α-ketoglutarate inhibited the synthesis of acetyl-CoA, thereby inhibiting the production of monacolin K. The addition of the above four kinds of substances in the citric acid cycle had a certain influence on the synthesis of monacolin K, which also proved the mutual influence and effect of the citric acid cycle and the synthesis of monacolin K (Figure 10B).

## Discussion

The PCA and supervised PLS-DA were used in the multivariate analyses for this study. The PCA was a statistical analysis method for mastering the main contradictions of things. It could analyze the main influencing factors from multiple things, reveal the essence of things, and simplify complex problems. The purpose of calculating the principal components was to project high-dimensional data into a lower-dimensional space to simplify the data and reduce the dimensionality. PCA is a multivariate statistical method that converts multiple variables into a few principal components (i.e., comprehensive variables) through dimensionality reduction technology. It is an unsupervised pattern recognition method.

Another common method of pattern recognition is the “supervised” pattern recognition method, which is a multivariate statistical method that first uses a set of samples or classifications with known results to establish a mathematical model and then uses several sets of independent and effective data to evaluate. PLS-DA has more obvious advantages than principal component analysis, that is, PLS has one more dependent variable “response” matrix than principal component analysis (PCA), so it has a predictive function.

The citric acid cycle is a key process in most plants, animals, fungi, and many bacteria (Enrique et al., 1996; Korla and Mitra, 2014; Wang et al., 2019). It consists of eight steps catalyzed by several enzymes. The citric acid cycle is initiated when acetyl-CoA reacts with oxaloacetate to form citrate. Acetyl-CoA is a common substance in the citric acid cycle and monacolin K biosynthetic pathways (Hajjaj et al., 1999). Accordingly, these two pathways may interact. Biotin participates extensively in the metabolic pathways of the three major nutrients as a coenzyme of acetyl-CoA. Under ammonium restriction, different proteins affecting the production of *Monascus* pigments mainly impact the glycolytic pathway, citric acid cycle, and fatty acid metabolic pathways.

A total of 6 differential metabolites related to the citric acid cycle were identified (fold-change > 2, VIP > 1, *p* < 0.05) between NG8 and G8, including fumaric acid, L-malic acid, oxoglutaric acid, *cis*-aconitic acid, citric acid, and thiamine pyrophosphate. The citric acid cycle acts as a central link between the metabolism of carbohydrates, lipids, and amino acids (Buchanan and Arnon, 1990). The metabolism of carbohydrates, lipids, and amino acids can produce acetyl-CoA, the initial substance in both the citric acid cycle and monacolin K biosynthetic pathways, as indicated above. Citric acid can promote the synthesis of monacolin K and influence the mycelial growth of *Monascus*. According to the previous experimental results, the addition of citric acid to the medium can increase the surface wrinkles of mycelium, and when the concentration of citric acid is 0.1%, the production of monacolin K is significantly increased, which is 2.7 times higher than that of the original medium. In addition, oxoglutaric acid is an intermediate metabolite in the citric acid cycle and a precursor for the synthesis of glutamic acid. In our study, the yield of monacolin K increased following the addition of glutamic acid. As shown in Table 3, the peak areas of fumaric acid, L-malic acid, *cis*-aconitic acid, citric acid, and thiamine pyrophosphate decreased after the addition of glutamic acid. We hypothesized that the addition of glutamic acid inhibited the citric acid cycle, and more acetyl-CoA was used in the synthesis of monacolin K.

**Table 3:** Differential metabolites of the citric acid cycle between G8-NG8.

**Compound name**	**Query mass**	**VIP**	**Delta (ppm)**	**Subclass**	**KEGG ID**	**Peak area in G8**	**Peak area in NG8**
Fumaric acid	114.9919	2.7177	103	Dicarboxylic acids and derivatives	C00122	222,940	346,850
L-Malic acid	133.0141	1.1576	1	Beta hydroxy acids and derivatives	C00149	6832	7701
Oxoglutaric acid	145.0146	1.5621	3	Gamma-keto acids and derivatives	C00026	12,330	16,923
*cis*-Aconitic acid	173.0074	1.5026	10	Tricarboxylic acids and derivatives	C00417	12,083	10,545
Citric acid	191.0201	1.5945	2	Tricarboxylic acids and derivatives	C00158	40,736	64,824
Thiamine pyrophosphate	424.0083	1.5915	69	Pyrimidines and pyrimidine derivatives	C00068	18,584	20,018

A total of 12 differential metabolites related to monacolin K were identified (fold change > 2, VIP > 1, *p* < 0.05) between NG8-G8-NG12-G12, including farnesyl pyrophosphate, natamycin, alpha-solanine, geranylgeranyl-PP, flavin mononucleotide, fumaric acid, L-malic acid, oxoglutaric acid, *cis*-aconitic acid, citric acid, dTDP-d-glucose, and 4,6-dideoxy-4-oxo-dTDP-d-glucose. Among these, farnesyl pyrophosphate, and geranylgeranyl-PP are non-fatty intermediate products in cholesterol synthesis (Takahashi, 1982). HMG-CoA reductase is an early rate-limiting enzyme in cholesterol synthesis, and monacolin K is a competitive inhibitor of HMG-CoA reductase (Chen and Hu, 2005; Niknejad et al., 2007). This is the first untargeted metabolomic profiling analysis of *Monascus* under different culture conditions by UPLC-Q-TOF-MS. In pathway analysis of differential metabolites using the KEGG database, we identified the citric acid cycle, biotin metabolism, alanine, aspartate, and glutamate metabolism as key pathways underlying differences among groups. Six differential metabolites related to the citric acid cycle were detected, including fumaric acid, L-malic acid, oxoglutaric acid, *cis*-aconitic acid, citric acid, and thiamine pyrophosphate. Five substances other than *cis*-aconitic acid decreased with the addition of glutamic acid. This study improves our understanding of secondary metabolites in *Monascus* in different culture conditions and benefits of *Monascus* in the food and pharmaceutical industries. Combined application of different “-omics” approaches, such as proteomics, metabolomics, and transcriptomics, can provide a more comprehensive view of the biochemical response in future studies.

## Data Availability Statement

The original contributions presented in the study are included in the article/supplementary materials, further inquiries can be directed to the corresponding author/s.

## Author Contributions

CZ, BS, and CW managed the project. NZ, MC, HW, and BW performed fungal culture, the secondary metabolites analysis, and the metabolomics results analysis in this work. HW and JS interpreted the analysis results and wrote the paper. All authors reviewed the manuscript.

## Conflict of Interest

The authors declare that the research was conducted in the absence of any commercial or financial relationships that could be construed as a potential conflict of interest.
